# Point-of-Care Ultrasound After Non-fatal Drowning in Rural Western Nepal: A Case Report

**DOI:** 10.5811/cpcem.50737

**Published:** 2026-03-29

**Authors:** Rochak Kansakar, Elijah J. Katz, Justin Zhao, Evan Weldon

**Affiliations:** * Bayalpata Hospital, Department of Emergency Medicine, Sanfebagar, Accham, Nepal; † University of Texas Southwestern Medical Center, Department of Emergency Medicine, Dallas, Texas

**Keywords:** point-of-care ultrasound, non-fatal drowning, rural emergency care, Nepal, case report

## Abstract

**Introduction:**

Drowning is a significant cause of death in Nepal, especially in rural areas. Identifying pulmonary edema is important for management of cases of non-fatal drowning, and while radiograph is the standard of care, point-of-care ultrasound (POCUS) offers a rapid, accessible alternative.

**Case Report:**

A 40-year-old woman presented to the emergency department after non-fatal drowning with respiratory distress and hypoxia. Chest radiograph was unavailable. Point-of-care ultrasound revealed diffuse B-lines consistent with pulmonary edema. She was stabilized and transported to a hospital with intensive care-level management.

**Conclusion:**

Point-of-care ultrasound enabled rapid identification of pulmonary edema and guided timely referral. In resource-limited settings, POCUS is a valuable tool for managing drowning victims when other diagnostics are unavailable.

## INTRODUCTION

Drowning represents a significant cause of injury and death in Nepal, with estimated mortality from drowning between 1.9 and 4.9 per 100,000 people.^1^,^2^ Children and men are the highest risk groups, and the burden from drowning is likely underestimated due to lack of surveillance systems and under-reporting.^1^,^3^ Rural areas in Nepal face further challenges from drowning because of greater access to bodies of water, flood susceptibility, and limited medical care.^3^

The hospital-based medical management of non-fatal drowning patients includes the assessment of respiratory and cardiovascular damage and the initiation of appropriate care, which can range from warming and low-flow oxygen to mechanical ventilation and extracorporeal membrane oxygenation.^4^ Best practice guidelines recommend pulse oximetry, arterial blood gases, chest radiograph, and basic metabolic panel as first-line diagnostics.^5^ However, in low-resource settings, point-of-care ultrasound (POCUS) represents a rapid, cost-effective, and versatile imaging modality with high sensitivity and specificity for relevant pathologic findings such as B-lines and pulmonary edema.^6^–^8^ Ultrasound can be used for risk-stratification of non-fatal drowning patients by monitoring for the presence and abundance of B-lines, which correlates directly with the presence and severity of pulmonary edema.^9^ Early ultrasound findings can guide appropriate management and referral decisions even in the absence of other imaging and labs.

This case report follows a 40-year-old female no-fatal drowning victim who was managed at Bayalpata Hospital in rural Western Nepal. The case highlights the utility of ultrasound in guiding management and risk stratification of patient care in austere medical centers.

## CASE REPORT

A 40-year-old female was brought to the emergency department (ED) following an accidental, non-fatal drowning in river water. The initial report stated the patient had been submerged for about one hour. The prolonged submersion in high-current waters with rocky surfaces resulted in facial and superficial injuries, as well as injuries to both lower limbs. On presentation the patient exhibited labored breathing and signs of respiratory distress, with room air oxygen saturation measuring 72% on pulse oximeter.

Physical examination revealed bilaterally decreased breath sounds, with crackles on auscultation over the infra-axillary regions. Her respiratory rate was 26 breaths per minute, and she appeared confused. Her Glasgow Coma Scale (GCS) score was 13: eye opening 4, verbal 4, and motor response 5. Blood pressure was 100/60 millimeters of mercury, temperature 94.5 °Fahrenheit, and heart rate 95 beats per minute. Supplemental oxygen was administered via face mask, and gradual rewarming was initiated. Bedside glucose measurement was 72 milligrams per deciliter (mg/dL) (reference range: 70–99 mg/dL).

Initial laboratory tests, including a complete blood count, renal function test, and liver function test, yielded results within normal limits. Arterial blood gases were not available at the hospital, and radiograph was temporarily unavailable due to a power outage. A POCUS was performed at the bedside using the handheld Butterfly iQ (Butterfly Network, Inc, Burlington MA) with the patient sitting semi-recumbent at a 45-degree angle. The lung zones assessed included the bilateral superior anterior, inferior anterior, superior lateral, and inferior lateral. Multiple B-lines, some confluent, were observed across most of the lung zones.

**Image 1:**
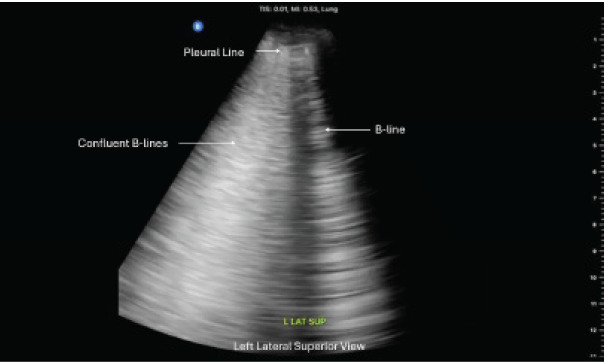
Point-of-care ultrasound of the left lung, in the left lateral superior view, showing confluent and individual B-lines in a non-fatal drowning patient.

**Image 2:**
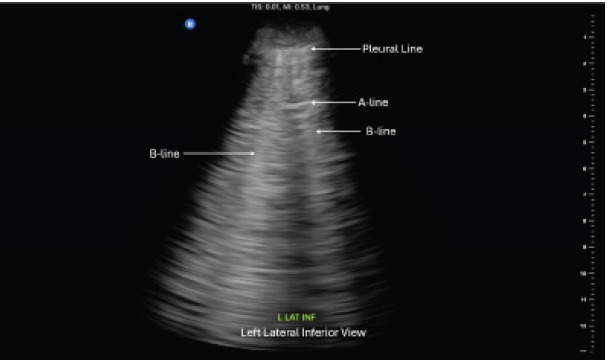
Point-of-care ultrasound of the left lung, in the left lateral inferior view, showing B-lines and partially obscured A-lines in a non-fatal drowning patient.

**Image 3:**
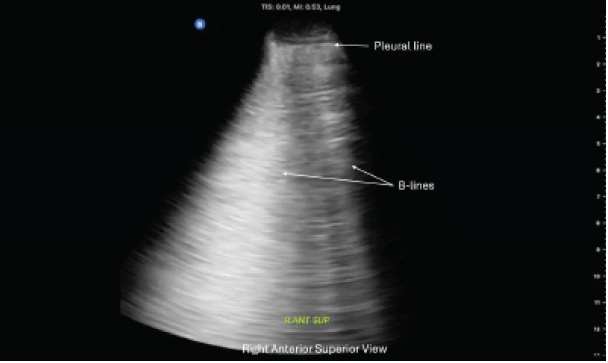
Point-of-care ultrasound of the right lung, in the right anterior superior view, showing numerous B-lines in a non-fatal drowning patient.

Ultrasound images from three representative zones are shown in Images 1–3. Additional findings revealed normal ventricular function and no evidence of pericardial effusion. The inferior vena cava was non-plethoric and had 50% collapsibility, suggesting adequate volume status. Given the clinical presentation of respiratory distress and the sonographic evidence of diffuse B-lines, a diagnosis of non-cardiogenic pulmonary edema secondary to the near-drowning event was made.

*CPC-EM Capsule*

What do we already know about this clinical entity?

*Non-fatal drowning patients require assessment of pulmonary edema to be appropriately managed and risk-stratified*.

What makes this presentation of disease reportable?

*This case demonstrates the successful use of point-of-care ultrasound (POCUS) in an austere medical environment as the sole imaging modality*.

What is the major learning point?

*POCUS can rapidly identify pulmonary edema after non-fatal drowning, enabling timely stabilization and transfer in low-resource settings*.

How might this improve emergency medicine practice?

*Expanding POCUS training and access in rural facilities can enhance early diagnosis and guide appropriate referral for non-fatal drowning victims*.

She was treated with supplemental oxygen via face mask. Gradual rewarming was initiated to correct hypothermia. Her blood pressure was maintained within a normal range with intravenous fluids. She was kept on non-invasive oxygenation via face mask at six liters per minute. Due to ongoing respiratory distress, positive pressure ventilation with bilevel positive airway pressure was considered; however, due to facial injuries around the nasal area, she could not tolerate it. Subsequently, the patient was sent by ambulance to another hospital for intensive care-level management.’

## DISCUSSION

Without access to radiograph or arterial blood gases, POCUS findings—specifically widespread B-lines suggesting severe pulmonary edema—helped guide the care of this patient and resulted in rapid transport to a center with mechanical ventilation capabilities. Pulmonary edema without clear signs of shock changed the patient from a grade 2 drowning victim, defined as rales in some pulmonary fields, to a grade 3. Grade 2 drowning patients can be managed in the ED with nasal cannula/non-rebreather oxygen, rewarming therapy, beta-agonists, and observation, all of which were available at the Bayalpata hospital.

In contrast, Grade 3 and above non-fatal drowning victims require intensive care-level management, arterial blood gas, advanced airway management and, in some cases, hemodynamic support, few of which were available.^10^ Given the evidence of likely aspiration and depressed mental status, supported by the abnormal POCUS findings, there was concern for potential deterioration requiring additional respiratory support.^11^ Therefore, the decision was made to rapidly transport the patient to Dadeldhura hospital by ambulance after the initiation of intravenous fluids, oxygen via nasal cannula, and rewarming therapy.

How does POCUS compare to radiograph in the detection of pulmonary edema? Ghauri et al reported that ultrasound has a sensitivity and specificity of 91.05% and 91.18%, respectively, for diagnosing pulmonary edema. In contrast, radiograph was shown to have a sensitivity of 60.16% and a specificity of 66.67%.^12^ It takes, on average, six minutes to accurately perform POCUS while the wait for an radiograph can vary widely in rural setting, if it is available at all.^13^ We believe that further ultrasound availability and training in rural areas of Nepal would be effective tools to enhance clinical management.

Limitations of this case report include a lack of follow-up after referral, initial radiograph confirmation for pulmonary edema, and additional supporting lab work. History, physical exam, basic labs, and POCUS were the driving factors of clinical management.

## CONCLUSION

This case highlights the role of point-of-care ultrasound in managing non-fatal drowning victims in resource-limited settings. In the absence of radiograph imaging, arterial blood gases, or advanced laboratory support, POCUS enabled the early detection of diffuse pulmonary edema and guided the decision to escalate care and refer to a higher center of care. The rapid bedside assessment provided risk-stratification, based on the diffuse and abundant B-lines, helping the patient receive appropriate care and reducing further deterioration. This case supports the growing body of evidence demonstrating the diagnostic utility of POCUS in austere environments. Broader implementation of ultrasound training and access in rural Nepal could improve the quality and timeliness of care for drowning patients.
